# A unique camouflaged mimarachnid planthopper from mid-Cretaceous Burmese amber

**DOI:** 10.1038/s41598-019-49414-4

**Published:** 2019-09-11

**Authors:** Tian Jiang, Jacek Szwedo, Bo Wang

**Affiliations:** 1 China University of Geosciences (Beijing), No. 29 Xueyuan Road, Haidian district, Beijing, 100083 China; 2 State Key Laboratory of Palaeobiology and Stratigraphy, Nanjing Institute of Geology and Palaeontology and Center for Excellence in Life and Paleoenvironment, Chinese Academy of Sciences, 39 East Beijing Road, Nanjing, 210008 China; 3 Laboratory of Evolutionary Entomology and Museum of Amber Inclusions, Department of Invertebrate Zoology and Parasitology, Faculty of Biology, University of Gdańsk, 59, Wita Stwosza St., PL80-308 Gdańsk, Poland; 4 Key Laboratory of Zoological Systematics and Evolution, Institute of Zoology, Chinese Academy of Sciences, Beijing, 100101 China

**Keywords:** Palaeontology, Palaeoecology

## Abstract

Predation is a major driving force for the evolution of functional forms. Avoidance of visual predators has resulted in different kinds of anti-predator defences, such as: camouflage, crypsis, disruptive coloration, and masquerade or mimesis. Camouflage is one of the forms involving shape, colouration, structure and behaviour when the visual pattern and orientation of an animal can determine whether it lives or dies. Inferring the behaviour and function of an ancient organism from its fossilised remains is a difficult task, but in many cases it closely resembles that of its descendants on uniformitarian grounds. Here we report and discuss examples of morphological and behavioural traits involving camouflage named recently as a flatoidinisation syndrome, shown by the inclusion of a planthopper in mid-Cretaceous Burmese amber. We found a new genus and species of an extinct Cretaceous planthopper family Mimarachnidae showing peculiar complex morphological adaptations to camouflage it on tree bark. Due to convergence, it resembles an unrelated tropiduchid planthopper from Eocene Baltic amber and also a modern representatives of the planthopper family Flatidae. Flattening of the body, the horizontal position of the tegmina at repose, tegmina with an undulating margin and elevated, wavy longitudinal veins, together with colouration and more sedentary behavioral traits enable these different insects to avoid predators. Our discovery reveals flatoidinisation syndrome in mid-Cretaceous Burmese amber which may provide insights into the processes of natural selection and evolution in this ancient forest.

## Introduction

“Everything changes, as Lyell^1^ knew from the fossil record, but everything is the same”. — Leigh Van Valen^2^.

The old adage “form follows function” is a guiding principle of functional morphology, a discipline, which is an interpretation of the function of an organism or organ system by reference to its shape, form and structure. Inferring the behaviour and function of an ancient organism from its fossilised remains is hard, but not impossible^3^, with general procedures for interpreting how organisms lived from their fossilized remains having been outlined^4^. The documentation of the wealth of information demonstrating behaviours of extant organisms extending far back in time and showing that the behaviour of extinct organisms closely resembles that of their descendants were recently summarized^5,6^.

Predation is a major driving force for the evolution of functional forms^7^. To avoid being detected by visual predators, different kinds of anti-predation defences have evolved, such as camouflage, crypsis, disruptive coloration, masquerade, or mimesis^8,9^. These anti-predator defences are adaptive to the organism’s surroundings, or in an aposematic organism, an inedible object that can affect the predation of visual-hunting predators^10–12^. Camouflage is one of the anti-predator defence functions involving shape, colouration, structure and behaviour^13^. Animals use camouflage to avoid detection or recognition by predators or prey^8,14,15^. Potential prey will benefit from being less visible or hidden nicely against their background, and gain more selective advantages to produce offspring (which will also inherit this trait)^14^. Among the group of predators competing for the same resources (prey), the predator that is not spotted easily has an advantage and makes more kills^16^. Prey and predators play the evolutionary game of hide-and-seek to survive, leading to the evolution of exquisite camouflage through natural selection.

Mimarachnidae is one of the extinct families of planthoppers (Fulgoroidea: Fulgoromorpha: Hemiptera), known exclusively from the Cretaceous. According to the former fossil records from the Berriasian-Barremian (ca. 145–125 Ma) deposits in Baissa (Buriatiya, Russia), early Cretaceous (ca. 140–120 Ma) Kaseki-kabe locality in Kuwajima (Japan), early Barremian deposit from Sierra del Montsec (north-eastern Spain), mid-Cretaceous Burmese amber and some undescribed specimens known from localities like Turga (central Siberia) of early Cretaceous, Khurilt (Mongolia) of Barremian or Aptian, Khetana (East Siberia) of Middle Albian, Kzyl-Zhar Hill (Kazakhstan) of Turonian^17–22^ the family was widespread from the equatorial to high latitude regions in the northern hemisphere in the Cretaceous period. First described from Lower Cretaceous compression fossils of Baissa^18^, the family is characterized by its simplified venation and setigerous metatibial pecten and hind leg amature^18–20^. Recently, several amber inclusions had been reported from Burmese amber^17,21,22^ which greatly increased the morphological disparity of the family, including species with different and peculiar morphological characters like giant size, elongated head, and a rostrum that exceeds the length of the body^17,21,22^.

**Figure 1:**
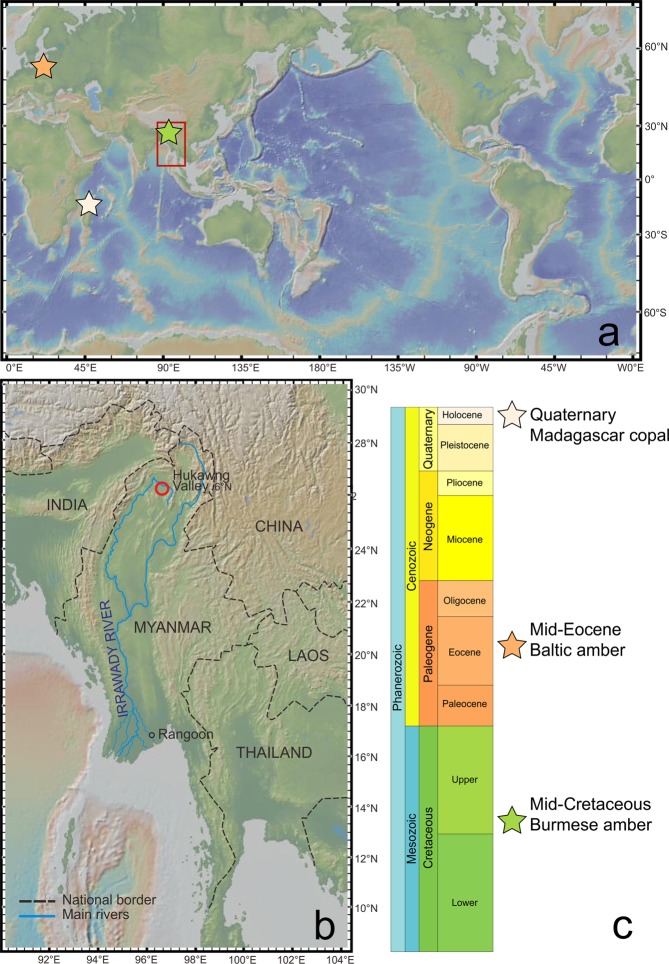
(**a**) Digital topographic map in the study area and adjacent region, derived from the Global Multi-Resolution Topography (GMRT) Synthesis (GeoMapApp: www.geomapapp.org/ CC BY/CC BY^83^). (**b**) World localities of fossils in which flatoidinisation syndrome is observed. (**c**) Stratigraphic column with fossil resins with inclusions showing flatoidinisation syndrome.

Herein, we report and discuss examples of another morphological and behavioural trait of camouflage, named recently as flatoidinisation syndrome^23^. This syndrome is now recognised from the family Mimarachnidae, preserved in mid-Cretaceous Burmese amber from northern Myanmar (Kachin State)^24–26^ (Fig. 1).

## Results

### Systematic palaeontology

Class Insecta Linnaeus, 1758.

Order Hemiptera Linnaeus, 1758.

Suborder Fulgoromorpha Evans, 1946.

Superfamily Fulgoroidea Latreille, 1807.

Family Mimarachnidae Shcherbakov, 2007.

### Genus *Mimaplax* gen. nov

LSID: urn:lsid:zoobank.org:act: 5DF955E9-883C-4E2D-9CD1-58BADB8B8311.

#### Type species

*Mimaplax ekrypsan* sp. nov. by present designation and monotypy.

#### Etymology

Generic name is derived from Ancient Greek words *mimos* for actor, mime, and *pláx* meaning anything flat and broad; making reference to body shape. Gender: neuter.

#### Diagnosis

Differs from other genera of Mimarachnidae in general appearance, being distinctly flattened; with membranous and translucent tegmen and widely rounded anterobasal angle, sinuate costal margin, and broad costal cell (wider than in *Chalicoridulum*; costal cell narrow in other congeners); head with vertex concave with lateral margins expanded above compound eyes (no such expansion in other genera with this character known); trigons not adjoining medially (trigons adjoining medially in *Burmissus*); pronotum and mesonotum with strongly elevated, cristate median carinae (median carinae not cristate in other genera); claval veins adjoining commissural margin (as in *Mimarachne*).

### Mimaplax ekrypsan sp. nov

**Figure 2:**
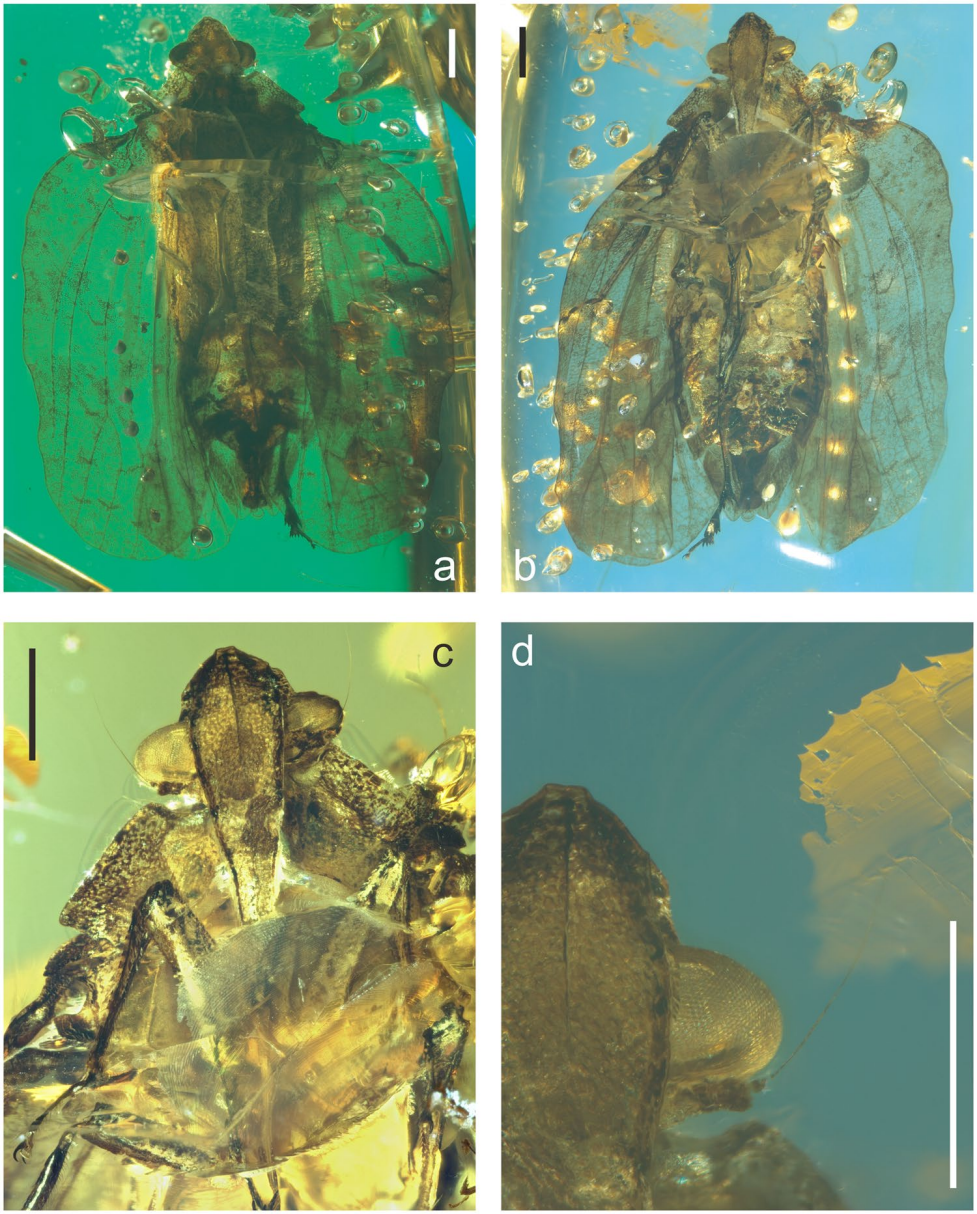
*Mimaplax ekrypsan* gen. et sp. nov. Photographs of amber inclusion: in dorsal view (**a**), in ventral view (**b**), head in anteroventral view (**c**), compound eye and antenna (**d**); scale bar 1 mm for all.

**Figure 3:**
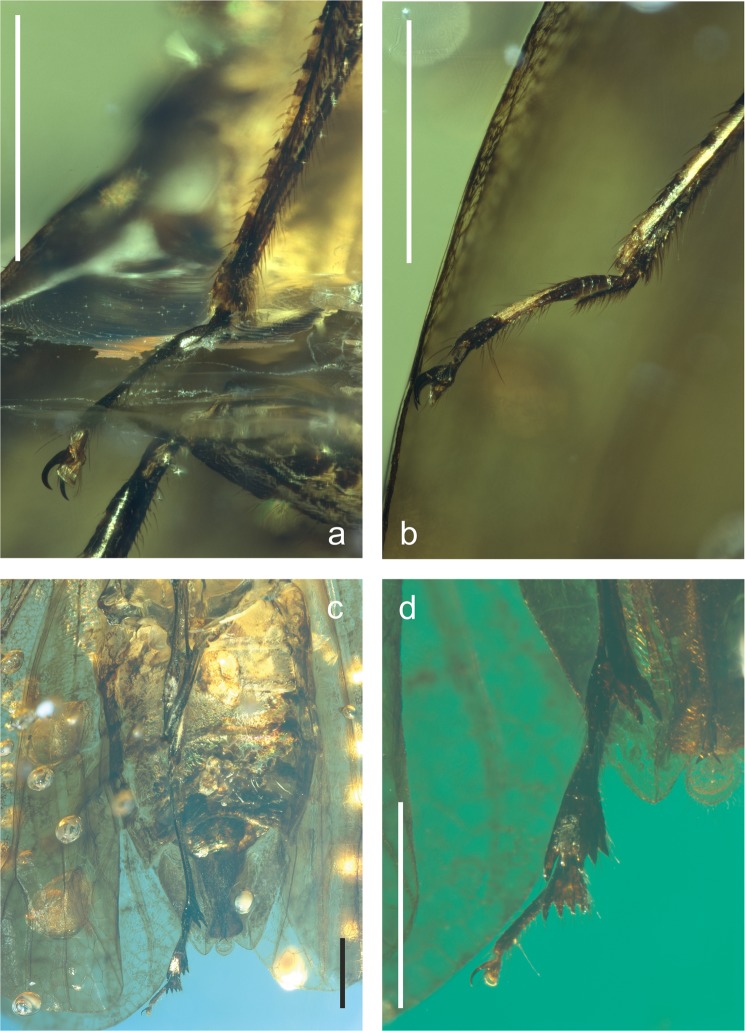
*Mimaplax ekrypsan* gen. et sp. nov. Photographs of legs and abdomen in ventral view (**a**), right protarsus (**b**), right mesotarsus (**c**), right metatarsus (**d**); scale bar 1 mm for all.

**Figure 4:**
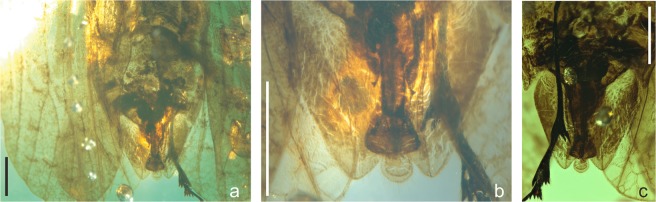
*Mimaplax ekrypsan* gen. et sp. nov. Photographs of abdomen and terminalia in dorsal view (**a**), detailed male terminalia in dorsal view (**b**), male terminalia in ventral view (**c**); scale bar 1 mm for all.

**Figure 5:**
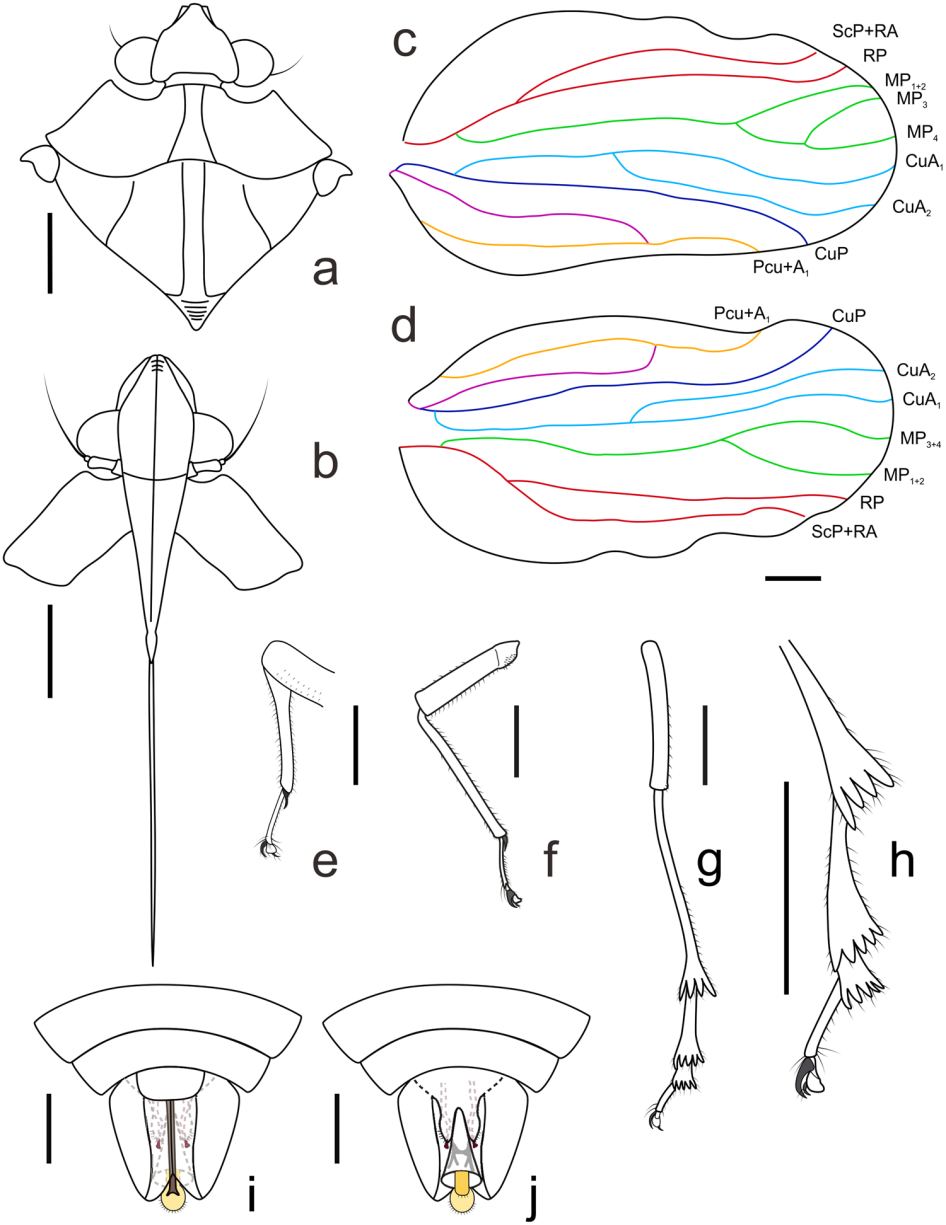
*Mimaplax ekrypsan* gen. et sp. nov. line drawings of relevant morphological structures: head and thorax (**a**), head and pronotum in anteroventral view (**b**), right tegmen with venation pattern labelled (**c**), left tegmen (**d**), proleg (**e**), mesoleg (**f**), metaleg (**g**), metatarsus (**h**), male terminalia in ventral view (**i)** and male terminalia in dorsal view (**j**); scale bar 1 mm for all. Abbreviations: ScP, subcosta posterior; RA, radius anterior; RP, radius posterior; MP, media posterior; CuA, cubitus anterior; CuP, cubitus posterior; Pcu, postcubitus; A, anal vein.

**Figure 6:**
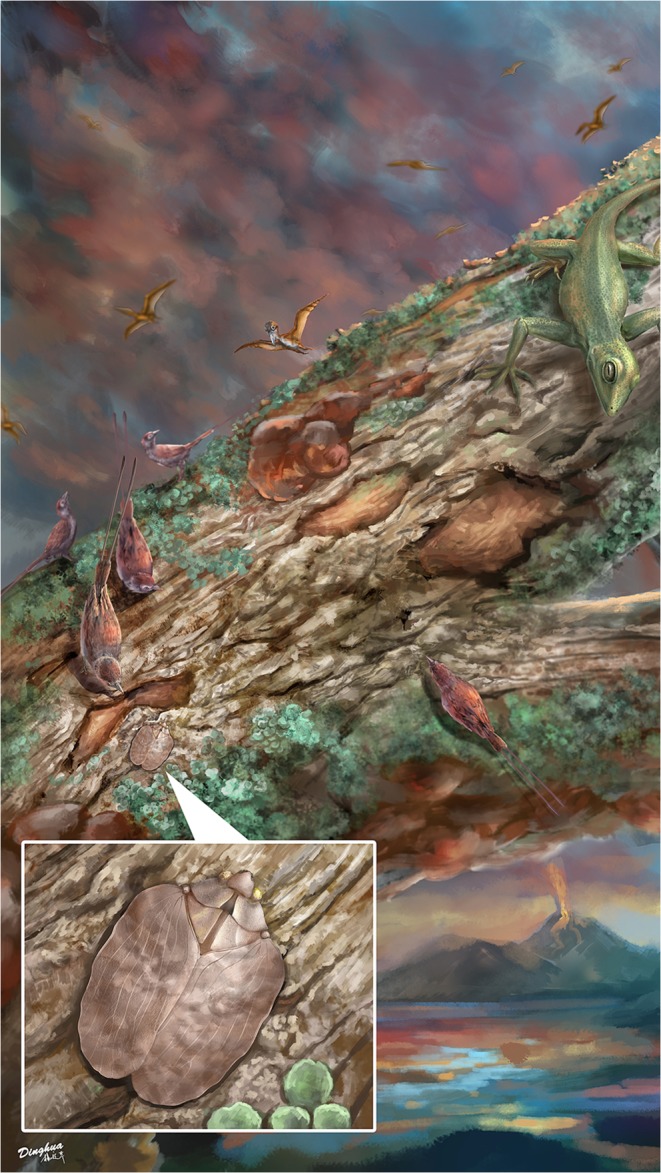
Reconstruction showing *Mimaplax ekrypsan* gen. et sp. nov. with surrounding habitat and possible predators from the mid-Cretaceous tropical forest in Burmese amber forest.

urn:lsid:zoobank.org:act: 7DF85E4E-F550-4098-8440-64D844B0416B.(Figures 2–6).

#### Etymology

Specific epithet is derived from Ancient Greek *ékrypsan*, meaning hidden one, and refers to cryptic characters of species.

#### Holotype

Burmese amber, elongate oval piece, 29 × 15 × 7 mm, weight 1.85 g. Specimen No. NIGP170539, deposited in Nanjing Institute of Geology and Palaeontology, Chinese Academy of Sciences, Nanjing. Holotype incomplete inclusion – head and abdomen partly preserved, including: pronotum, mesonotum, left tegmen, right tegmen, hind wings, fore legs, mid legs and hind legs. Syninclusions: nymph of Hemiptera: Fulgoroidea: Neazoniidae, 0.8 mm long.

#### Locality and horizon

Burmese amber, Noije Bum hill, Hukawng Valley, Kachin State, northern Myanmar^17,25,26^. Terminal Aptian/earliest Cenomanian^27,28^ (Fig. 1).

#### Diagnosis

Rostrum reaching metacoxae. Tegmen with branch ScP + RA not reaching margin nor RP, stem MP with two terminal branches. Pro- and mesolegs with basi- and midtarsomeres’ plantar surfaces covered with brush of setae; metatibio-metatarsal formula (apical teeth) 4: 5: 5. Male anal tube widened apically, longer ventrally than dorsally; anal cercus subquadrate, anal style roundly lingulate with long apical setae. Gonostyle long and narrow, but distinctly shorter than aedeagus, S-shaped, widened apically. Aedeagus long, tube-like, forked apically, periandrium not visible.

*Description*. (see appendix).

## Discussion

Camouflage is one of the most common anti-predator strategies in nature^9,10,29–32^. It is the art of concealment and it can be achieved in many different ways: matching the background like self-decoration or debris-carrying, disruptive colouration for masking edge information, masquerading as a non-target object like leaf mimesis, or actively changing colour and pattern^10,13,14,32–37^. Exceptionally preserved fossils can show examples of camouflage. The oldest record of cryptic coloration comes from the Carboniferous^38^. Various forms of these phenomena can be found among Jurassic insects from the Daohugou biota among the Palaeontinidae (Hemiptera)^39–42^, Orthoptera^43,44^, Mecoptera^45^ and Neuroptera^46^. Cretaceous ambers reveal several spectacular examples of camouflage in small predatory insects^47,48^ including chrysopoid larvae (green lacewings), myrmeleontoid larvae (split-footed lacewings and owlflies), and reduviids (assassin bugs). Another example of camouflage comes from archostematan beetles^49,50^.

Terms and definitions relevant to visual camouflage are listed^14^ where camouflage is treated in a wider sense, meaning all strategies involved in concealment for prevention of detection and recognition. Crypsis is a narrower term covering mechanisms initially preventing detection. It involves at least shape, colour, and colour pattern. However, a cryptic individual must also solve the major problem of body contour. For homogeneous backgrounds the cryptic coloration can efficiently increase the difficulty of detection and recognition by visual hunting predators, but the predation risk will increase in heterogeneous habitats where a background matching solution performs poorly. One solution is to combine colouration and shape in disruptive patterns, most widely used as disruptive coloration, a visual breaking up of the body outline so that parts of it appear to fade separately into the background^36^. Another way of minimizing contour cues involves actually or apparently reducing any tell-tale shadows, accomplished through a dorsoventral flattening, often in combination with lateral flaps or various irregular body protuberances that bridge the gap between body and substrate, referring to countershading in some animals^31,51–55^.

**Figure 7:**
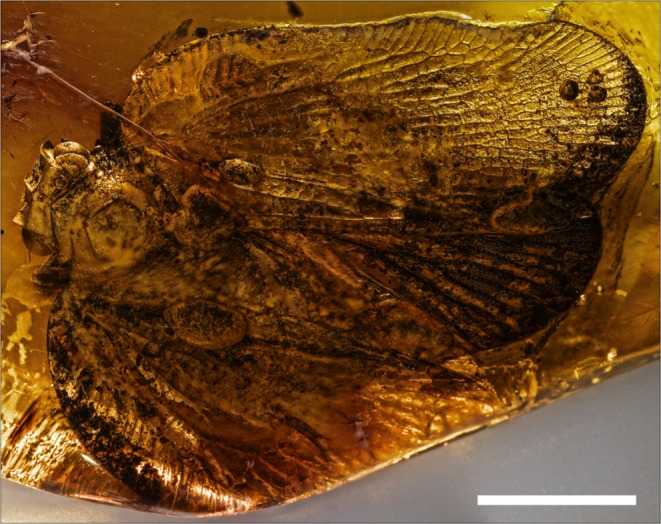
*Gedanotropis sontagae* Szwedo et Stroiński, 2017, Tropiduchidae, Eocene Baltic amber – fossil planthopper showing flatoidinisation syndrome; scale bar 5 mm.

In our case, flatoidinisation syndrome was proposed to represent a specialised, complex camouflage, uniting shape, colour and behaviour^23^. The name of the syndrome is derived from morphological similarity to some representatives of the planthopper family Flatidae (Hemiptera: Fulgoroidea) and subfamily Flatoidinae. These groups contain taxa that are in most cases distinctly dorsoventrally flattened, sometimes very strongly flattened, with shapes and colouration enabling them to be almost invisible on tree bark, or on lichens and other plants covering the bark of trees in the tropical and subtropical zones^56–62^. Flatoidinae is one of subfamilies of flatid planthoppers, currently the subfamily comprises 25 genera and 225 species^59^. The flatoidinisation syndrome is presented among them to various extent, with the most spectacular forms among taxa inhabiting Madagascar^61–63^. Some elements of flatoidinisation syndrome (flattening of body with wings held tectiform or horizontally with camouflage or disruptive coloration) can be observed in unrelated planthoppers of the families Eurybrachidae, Lophopidae, Ricaniidae and Fulgoridae^23,56^. However, the most complete and complex flatoidinisation syndrome in morphology and behaviour is presented by Flatoidinae flatids. Among fossils the syndrome was described first in a representative of the fossil genus *Gedanotropis* Szwedo et Stroiński, 2017, from Eocene Baltic amber, belonging to the planthopper family Tropiduchidae^23^. Similar to representatives of flatoidine Flatidae^60–62^, the shape of the tegmina of *Gedanotropis* is subquadrate, the anterior portion of the costal margin being strongly curved and shifted anteriad, the costal margin is undulate, the tegmina are held flat, and longitudinal veins are polychotomous (Fig. 7). This shape suggests that the insect was hiding on tree trunks, sitting flat on the bark, reducing shadows with lateral undulations of the tegminal costal margin. The colour pattern of *Gedanotropis* remains unknown, but very probably it presented some camouflage colouration.

The flatoidinisation syndrome is developed also in the newly described mid-Cretaceous genus *Mimaplax*. Contrary to previously described Mimarachnidae^17–22^, it is flattened dorsoventrally, with widened tegmina held flat and with undulate margins. The main veins are not polychotomous (multiforked), but irregularly wavy, and elevated, probably resembling the texture of the background. Also the elevated, cristate median carinae of the pronotum and mesonotum are devices of concealment on tree bark. The head capsule, flattened, with concave disc, and lateral margins elevated above the eyes, resembles the situation in modern representatives of Flatoidinae flatids. In addition, traces of cryptic coloration are preserved in remnants of irregular darker patches, bands and spots in *Mimaplax* (Figs 2 and 6).

What are the reasons for such sophisticated defence mechanisms? The answer is simple – pressure from predators. The behaviour of Flatoidinae from Madagascar, where the most numerous and bizarre forms are to be found (12 genera with 39 species^58,59,63^) is virtually unknown, but among potential predators of these relatively huge hoppers several groups of vertebrates should be taken into consideration: lizards, chameleons, birds and small mammals^64^.

The same pressure from predators could have resulted in flatoidinisation syndrome in *Mimaplax* within the forests of the mid-Cretaceous equatorial area of the West Burma terrane and adjacent islands. A warm, humid, nearshore marine setting with high species diversity has been proposed for the amber locality^17,24–26,65,66^. Potential predators with good visual ability to distinguished cryptic prey have been reported from the amber locality, like small non-avian theropod dinosaurs^67^, enanthiornithid birds^68–70^ and various lizards^71^. These creatures could penetrate tree trunks, branches, twigs and tree canopies in search of prey (Fig. 6). Small theropods and enanthiornithids (like today’s birds) likely had tetrachromatic vision enhanced by a suite of oil-droplet filters^72–74^ which made them very efficient in locating and recognising potential insect prey^75^. In this context, background pattern matching may be insufficient to conceal objects because of edge information. A ruffled outline of the body better conceals the insect than a straight boundary outline^36^. Colouration with patches touching the outline and differentially blending into the background, disrupt the continuity of extended edges, or translucence mixed with solid patches, may break up the continuity of the outline^36,76–79^. Vertebrate visual systems perform more effectively when detecting straight boundaries compared to curvilinear boundaries^80^, and such combination of shape and coloration, were likely supplemented by behaviour^55,79^. The probability of an individual being attacked by a predator is dependent on the level of matching an animal has to its background, as seen through the eyes of the key predators.

Ultimately, *Mimaplax ekrypsan* gen. et sp. nov., offers an unprecedented opportunity to observe morphological adaptations including sophisticated camouflage leading to flatoidinisation syndrome, providing exceptional and unexpected insights into the evolution of the Cretaceous Mimarachnidae.

## Methods

The specimen was prepared in the Laboratory of Evolutionary Entomology and Museum of Amber Inclusions, University of Gdańsk, Poland, and was observed under a stereoscopic microscope with varying illumination and filters to increase contrast of pigmentation and morphological details. Photographs were taken using a Zeiss Stereo Discovery V.16 microscope system with Zen software, in the Nanjing Institute of Geology and Palaeontology, Chinese Academy of Sciences. All images are digitally stacked photomicrographic composites of more than 50 individual focal planes obtained using the free software Combine ZP for a better illustration of 3D structures. The line drawings were prepared with Nikon microscope (SMZ1000) with a drawing tube attached, photographs and drawings were adjusted using CorelDraw X8 and CorelPhoto-Paint X8 packages. The specimen NIGP170539 is housed at the Nanjing Institute of Geology and Palaeontology, Chinese Academy of Sciences (NIGPAS). The nomenclature of the wing venation used in this paper is based on the general scheme for the Hemiptera^81,82^.

## Supplementary information

Appendix 1 Jiang, Szwedo & Wang: A unique camouflaged mimarachnid planthopper
