# Supplementary material for: A unique camouflaged mimarachnid planthopper from mid-Cretaceous Burmese amber
Source: Sci Rep. 2019 Sep 11;9:13112. doi: 10.1038/s41598-019-49414-4 (PMC6739471; doi:10.1038/s41598-019-49414-4)
Supplement: Supplementary file 1 — Appendix 1 Jiang, Szwedo & Wang: A unique camouflaged mimarachnid planthopper [file 41598_2019_49414_MOESM1_ESM.pdf]

A unique camouflaged mimarachnid planthopper from mid-Cretaceous Burmese amber

Tian Jiang, Jacek Szwedo, Bo Wang

Appendix 1

Systematic palaeontology

(with detailed descriptions)

Class Insecta Linnaeus, 1758

Order Hemiptera Linnaeus, 1758

Suborder Fulgoromorpha Evans, 1946

Superfamily Fulgoroidea Latreille, 1807

Family Mimarachnidae Shcherbakov, 2007

Diagnostic characters. Pronotum and mesonotum with double median carinae; fore wings (tegmina) and hind wings with poor vein branching and meshwork of crossveins; tegmen with ScP+R forked basally, branch RP elevated more prominently than branch ScP+RA; basal cell with weak 'arculus' (basal portion of branch CuA); hindwing with single stem M; metatibia without lateral spines.

Range. Cretaceous (Valanginian–Turonian).

Genus *Mimaplax* gen. nov.

LSID: urn:lsid:zoobank.org:act: 5DF955E9-883C-4E2D-9CD1-58BADB8B8311

Type species. *Mimaplax ekrypsan* sp. nov. by present designation and monotypy.

Etymology. Generic name is derived from Ancient Greek word mimos (μῖμος, μῖμου, μῖμό) – actor, mime, and pláx, plákós (πλάξ , πλάκός) meaning anything flat and broad and refers to

the shape of the inclusion. Gender: neuter.

Diagnosis. Differs from other genera of Mimarachnidae in general appearance, being distinctly flattened; with membranous and translucent tegmen and widely rounded anterobasal angle, sinuate costal margin, and broad costal cell (wider than in *Chalicoridulum*; costal cell narrow in other congeners); head with vertex concave with lateral margins expanded above compound eyes (no such expansion in other genera with this character known); trigons not adjoining medially (trigons adjoining medially in *Burmissus*); pronotum and mesonotum with strongly elevated, cristate median carinae (median carinae not cristate in other genera); claval veins adjoining commissural margin (as in *Mimarachne*).

Mimaplax ekrypsan **sp. nov.**
urn:lsid:zoobank.org:act: 7DF85E4E-F550-4098-8440-64D844B0416B
(Figures 2-6)

Etymology. Specific epithet is derived from Ancient Greek ékrypsan (ἔκρυψαν) meaning the one hidden and refers to cryptic characters of the species

Holotype. Burmese amber, elongate oval piece, 29×15×7 mm, weight 1.85 g. Specimen No. NIGP170539 deposited in Nanjing Institute of Geology and Palaeontology, Chinese Academy of Sciences, Nanjing (NIGPAS). Holotype; incomplete inclusion - head and abdomen partly preserved, including: pronotum, mesonotum, left tegmen, right tegmen, hindwings, forelegs, midlegs and hindlegs. Syninclusions: nymph of Hemiptera: Fulgoroidea: Neazoniidae, 0.8 mm long.
Locality and horizon. Burmese amber, Noije Bum Hill, Hukawng Valley, Kachin State, northern Myanmar[17, 24-26]. Terminal Aptian/earliest Cenomanian[27, 28] (Fig. 1).

Diagnosis. Rostrum reaching metacoxae. Tegmen with branch ScP+RA not reaching margin nor RP, stem MP with two terminal branches. Pro- and mesolegs with basi- and

midtarsomeres' plantar surfaces covered with brush of setae; metatibio-metatarsal formula (apical teeth) 4: 5: 5. Male anal tube widened apically, longer ventrally than dorsally; anal cercus subquadrate, anal style roundly lingulate with long apical setae. Gonostyle long and narrow, but distinctly shorter than aedeagus, S-shaped, widened apically. Aedeagus long, tube-like, forked apically, periandrium not visible.

Description. Body with preserved wings and head 11.4 mm long. Head including compound eyes 2.1 mm wide, narrower than the pronotum; vertex triangular, lateral edges slightly converging anteriorly, posteriorly reaching pronotal disc anterior margins, 1.3 mm in length at mid line. Trigons distinct in ventral view; frons punctate, subhexagonal, 1.2 mm long near mid line, 0.8 mm wide at widest point proximad of compound eyes anteriorly; median carina faint before reaching clypeal suture proximad of antenna. Clypeus punctate, slightly convex; lateral margin carinate, converging rearward, median carina more distinct in rear part; posterior portion of clypeus unclear, covered by cracks. Antenna 1.2 mm long.

Thorax. Pronotum subhexagonal, 1.1 mm long near mid line, 3.6 mm wide; median carinae doubled, strongly elevated; disc of pronotum separated by shallow furrows; puncturation slightly more distinct and larger on lateral lobes of pronotum than on disc.

Mesonotum subpentagonal, 2.0 mm wide, 3.7 mm long; median carinae doubled, strongly elevated; lateral carinations distinct, sinuate, slightly elevated, faint before converging with anterior margin.

Tegula subquadrilateral, wider (0.8 mm) than long (0.6 mm) near mid line, punctate, slightly upturned, convex slightly in mid line level and carinate distinctly.

Tegmen 9.0 mm long, 4.0 mm wide, membranous, translucent with distinct carinate longitudinal veins and polygonal net of transverse veinlets; veins and margins not smooth, sinuate; Basal cell (R) nearly 3 times as long as wide, left tegmen (L) basal cell slightly narrower. Stem ScP+R short, leaving basal cell basad of stem MP; branch ScP+RA sinuate, gradually approaching costal margin and becoming subparallel to posterior margin; branch RP subparallel to branch ScP+RA. Stem MP curved at base then subparallel to branch RP, forking first apicad of terminal point of stem Pcu+A_1; branch MP_{1+2} single, branch MP_{3+4} (R)

forked once at level of claval apex, reaching margin with 2 terminals, branch MP_{3+4} (L) single. Stem CuA forked proximad of junction of claval veins Pcu and A_1; branch CuA_2strongly curved at base and approaching claval vein CuP closely, then curved backwards and becoming subparallel to branch CuA_1. Claval vein CuP almost straight and grooved in foreside, and then curved and bulging, reaching margin apicad of posteroapical angle; claval veins Pcu and A_1 fused apicad of stem ScP+R forking, basad of stem MP forking; A_1 (L) strongly curved apically before fusing with Pcu, Pcu (L) strongly sinuate; stem Pcu+A_1 relatively short, curved mediad. Costal area broad, costal cell as wide as costal area, closing with ScP+RA and reaching margin, with net of polygonal cells; Cell C1 open and narrow; Cell C3 open, widen in basal portion; cell C5 open, narrow, curved and lanceolate.

Legs. Profemur flattened, with short setae on lateral side; protibia (1.6 mm) carinate, flattened, longer than protarsus, margins of both provided with rows of short, delicate setae; protibial spur preserved, protarsus two-segmented. Mesoleg 5.0 mm long, mesocoxa short and half covered with short and broad setae; mesofemur (1.7 mm long) carinate and flattened, shorter than mesotibia (2.3 mm), mesotibial spur preserved, mesotibial apex with short hairs; mesotarsus (1.0 mm) with rows of lateral short setae. Tarsomeres of proleg and mesoleg of similar length, tarsal claws and arolium distinct, and covered with long setae. Metaleg 6.5 mm long, metafemur 1.9 mm long, shorter than metatibia, both with uniseriate, short setae; metatibia 2.7 mm long, with 4 apical teeth and rows of short setae; metatarsus 2.0 mm long, basimetatarsomere 1.08 mm long, about as long as combined length of mid and apical metatarsomeres, with row of 5 setigerous apical teeth; midmetatarsomere 0.45 mm long, with row of 5 setigerous apical teeth; apical metatarsomere (0.63 mm) longer than midmetatarsomere, tarsal claws and wide arolium developed, provided with long setae.

Abdomen compressed due to preservation. Male terminalia (Figs. 4, 5E-F) with two symmetrical lobes covering most of genital structures in ventral view; pygofer lobes foliaceous, membranous, translucent, with carinae in mid line; inner structure of pygofer covered by 8th sternite. Anal tube fused to pygofer in dorsal view, with apex distinct and circular, and two anal styles protruding. Anal styles membranous, with a row of long setae apically. Styles double, attached to aedeagus by kind of Y-shaped connective, elongate, and

extended to both sides of anal tube separately, sclerotized in apical portion. Aedeagus elongate, reaching anal style apex apically, with distal flap-like flagellum.

Additional references

(not listed in the main text)

Evans, J.W. A natural classification of leaf-hoppers (Jassoidea, Homoptera). Part 1. External morphology and systematic position. *T. Roy. Ent. Soc. London* **96**, 47–60. doi:10.1111/j.1365-2311.1946.tb00442.x (1946).

Latreille, P.A. *Genera Crustaceorum et Insectorum, secundum ordinem naturalem in familias disposita, iconibus exemplisqueplurimis explicata*. 3, 1–258 (Parisiis et Argentorati, apud Amand Koenig, bibliopolam, 1807).

Linnaeus, C. *Systema Naturae per Regna tria Naturae, secundum classes, ordines, genera, species, cum characteribus, differentiis, synonymis, locis. Editio decima, reformata* 1. 1–824 (Impensis Direct. Laurentii Salvii, Holmiae, 1758).
